# Analysis of *MDM2* Amplification: Next-Generation Sequencing of Patients With Diverse Malignancies

**DOI:** 10.1200/PO.17.00235

**Published:** 2018-07-13

**Authors:** Shumei Kato, Jeffrey S. Ross, Laurie Gay, Farshid Dayyani, Jason Roszik, Vivek Subbiah, Razelle Kurzrock

**Affiliations:** **Shumei Kato** and **Razelle Kurzrock**, University of California, San Diego, Moores Cancer Center, La Jolla; **Farshid Dayyani**, University of California, Irvine, Orange, CA; **Jeffrey S. Ross** and **Laurie Gay**, Foundation Medicine, Boston, MA; and **Jason Roszik** and **Vivek Subbiah**, The University of Texas MD Anderson Cancer Center, Houston, TX.

## Abstract

**Purpose:**

*MDM2* amplification can promote tumorigenesis directly or indirectly through p53 inhibition. *MDM2* has increasing clinical relevance because inhibitors are under evaluation in clinical trials, and *MDM2* amplification is a possible genomic correlate of accelerated progression, known as hyperprogression, after anti–PD-1/PD-L1 immunotherapy. We used next-generation sequencing (NGS) to ascertain *MDM2* amplification status across a large number of diverse cancers.

**Methods:**

We interrogated the molecular profiles of 102,878 patients with diverse malignancies for *MDM2* amplification and co-altered genes using clinical-grade NGS (182 to 465 genes).

**Results:**

*MDM2* amplification occurred in 3.5% of patients (3,650 of 102,878). The majority of tumor types had a small subset of patients with *MDM2* amplification. Most of these patients (99.0% [3,613/3,650]) had co-alterations that accompanied *MDM2* amplification. Various pathways, including those related to tyrosine kinase (37.9% [1,385 of 3,650]), *PI3K* signaling (25.4% [926 of 3,650]), *TP53* (24.9% [910 of 3,650]), and *MAPK* signaling (23.6% [863 of 3,650]), were involved. Although infrequent, mismatch repair genes and *PD-L1* amplification also were co-altered (2.2% [79 of 3,650]). Most patients (97.6% [3,563 of 3,650]) had one or more co-alterations potentially targetable with either a Food and Drug Administration–approved or investigational agent. *MDM2* amplifications were less frequently associated with high tumor mutation burden compared with the *MDM2* wild-type population (2.9% *v* 6.5%; *P* < .001). An illustrative patient who harbored *MDM2* amplification and experienced hyperprogression with an immune checkpoint inhibitor is presented.

**Conclusion:**

*MDM2* amplification was found in 3.5% of 102,878 patients, 97.6% of whom harbored genomic co-alterations that were potentially targetable. This study suggests that a small subset of most tumor types have *MDM2* amplification as well as pharmacologically tractable co-alterations.

## INTRODUCTION

The *MDM2* proto-oncogene encodes a nuclear localized E3 ubiquitin ligase. The core function of MDM2 is to inhibit the tumor suppressor p53, which is critical for regulating genes involved in DNA repair, cell cycle, senescence, and apoptosis. When amplified, *MDM2* facilitates proteasomal degradation of p53, which promotes tumorigenesis.^1-3^ *MDM2* amplification has been reported in multiple tumor types^4-6^ and is a hallmark of tumorigenesis.^3^ In certain tumor types, such as glioblastoma and well-differentiated liposarcoma, *MDM2* amplification and *TP53* alterations are mutually exclusive,^4,5^ which is consistent with the inhibitory function of MDM2. However, in other tumors (eg, osteosarcoma, esophageal cancer), *MDM2* amplification and *TP53* alterations co-occur.^5,6^

Preclinical studies have suggested noncanonical p53-independent roles for MDM2. For instance, an in vitro study that used an MDM2-overexpressed/*TP53* wild-type cancer cell line revealed a potential role for MDM2 in suppressing senescence in a TP53-independent fashion.^7^ Moreover, *MDM2*-amplified/*TP53*-null mice have a higher incidence of spontaneous tumorigenesis than *TP53*-null mice (without *MDM2* amplification).^8^ Among several potential noncanonical roles for MDM2, a functional angiogenesis effect has been proposed.^9^ Indeed, a preclinical study showed that under hypoxic conditions, MDM2-overexpressed/*TP53*-null cancer cells produce vascular endothelial growth factor (VEGF) mRNA at higher levels than MDM2-negative/*TP53*-null cells. Hypoxia induces translocation of MDM2 from the nucleus to the cytoplasm, and subsequent binding of the MDM2 C-terminal domain to the 3′untranslated region of VEGF mRNA increases mRNA stability and translation.^10^ In addition, suppression of MDM2 activity with a small-molecule inhibitor leads to decreased hypoxia-inducible factor 1α and VEGF expression, which support a role for MDM2 in angiogenesis.^11^ *TP5*3 mutations also lead to increased VEGF-A expression^12,13^ and have been associated with increased responsiveness to VEGF/VEGF receptor inhibitor therapy.^14-16^ Taken together, these data suggest that MDM2 promotes angiogenesis through either inhibition of p53 or mechanisms independent of p53.

An understanding of *MDM2* amplification status has clinical relevance for patients with cancer because MDM2 inhibitors are in early-phase clinical development (Data Supplemental). Although preliminary results demonstrated no responses to MDM2 inhibitors among unselected patients,^17-19^ responses occurred in wild-type *TP53* liposarcoma (MK-8242; response rate [RR], 11.1% [three of 27 patients]) or melanoma (AMG232; RR, 28.6% [six of 21 patients]).^20,21^

*MDM2* amplification also has been implicated as a potential marker for accelerated tumor growth with receipt of immune checkpoint inhibitors.^22^ This phenomenon is called hyperprogression and affects approximately 9% of patients who receive PD-1/PD-L1 inhibitors.^23^ Hyperprogression has been defined as a time to treatment failure < 2 months from checkpoint inhibitor initiation, a > 50% increase in tumor burden compared with pre-immunotherapy imaging, and a more than two-fold increase in progression pace.^22^ To date, accelerated progression after anti–PD-1/PD-L1 agents has been reported by at least four groups.^22-25^ Although the mechanisms that mediate this phenomenon remain unclear, we and others have demonstrated that *MDM2* family gene amplifications and *EGFR* alterations correlate with hyperprogression.^22,25^

Given the clinical importance of *MDM2*, we describe the landscape of cancer types that harbor *MDM2* amplification and evaluate the comprehensive genomic profiles of 102,878 tumors from patients with malignancies. An illustrative patient with *MDM2* amplification that demonstrated hyperprogression after checkpoint blockade is presented.

## METHODS

### Patients

We explored the *MDM2* amplification status of patients with diverse malignances who were referred for comprehensive next-generation sequencing (NGS) from June 2012 through December 2016 (N = 102,878; Table 1; Data Supplement). A de-identified database with cancer diagnoses and molecular profiling results was available. This study was performed in accordance with the guidelines of the University of California, San Diego, institutional review board with regard to analysis and consent.

**Table 1.**
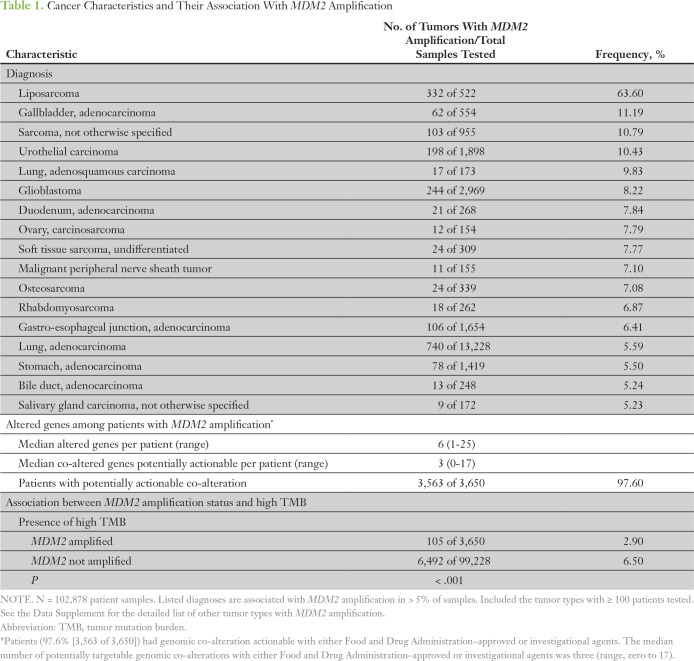
Cancer Characteristics and Their Association With *MDM2* Amplification

### Tissue Samples and Mutational Analysis

Tumors were provided as formalin-fixed, paraffin-embedded samples and evaluated by NGS in a Clinical Laboratory Improvement Amendments–certified laboratory (Foundation Medicine, Cambridge, MA). The methods used for NGS have been validated and previously reported.^26-29^ DNA was adaptor ligated, and hybrid capture was performed for all coding exons of 182 to 465 cancer-related genes plus select introns from 14 to 31 genes frequently rearranged in cancer (Data Supplement). For samples in which RNA was available, targeted RNA sequencing was performed for rearrangement analysis in 333 genes (Data Supplement). Sequencing was performed with an average sequencing depth of coverage > 250×, with > 100× at > 99% of exons. Somatic mutations are identified with 99% specificity and > 99% sensitivity for base substitutions at ≥ 5% mutant allele frequency and > 95% sensitivity for copy number alterations. Gene amplification is reported at eight or more copies above ploidy, with six or more copies considered equivocal. The exception is *ERRB2* for which five or more copies are considered equivocal amplification.^28,29^ Only characterized genomic alterations (not variants of unknown significance) were curated for all analyses except those for tumor mutation burden (TMB).

### TMB

TMB was calculated on the basis of the total number of mutations counted per megabase.^30^ Noncoding alterations, alterations reported as known somatic alterations in Catalog of Somatic Mutations in Cancer, truncations in tumor suppressor genes, and alterations predicted to be germline were not counted. High TMB was defined as ≥ 20 mutations/megabase.

### Cancer Genomic Data Through Publicly Available Data Sets

*MDM2* amplification status was also curated from the Genomics Evidence Neoplasia Information Exchange (GENIE) accessed in July 2017.^31-33^

### End Points, Statistical Methods, and Case Study

Descriptive statistics were used to summarize the cancer diagnoses and genomic alterations identified in the data set. Statistical analyses were carried out using GraphPad Prism 7 software (GraphPad Software, La Jolla, CA). A patient with *MDM2* amplification who experienced hyperprogression while receiving an immune checkpoint inhibitor is presented.

## RESULTS

### Evaluation of *MDM2* Amplification Among Diverse Cancers

**Fig 1.**
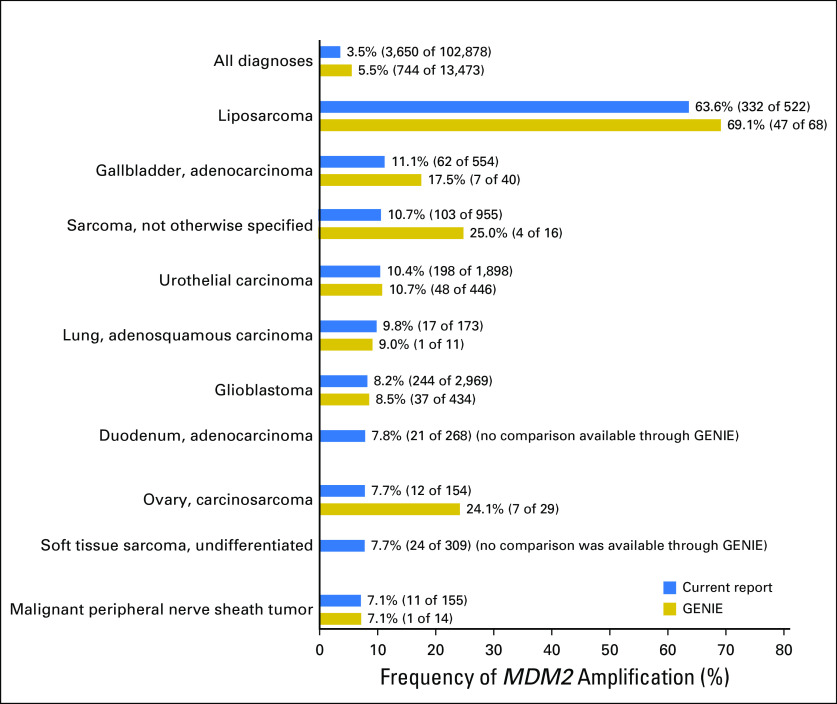
Comparison of rate of *MDM2* amplification in the current report (N = 102,878 samples) versus in Genomics Evidence Neoplasia Information Exchange (GENIE; N = 13,473 samples).^31^ Data from GENIE were obtained as previously described.^31^ The 10 most common diagnoses that harbor *MDM2* amplification from the current report were selected for the comparison. *MDM2* amplifications were seen in 3.5% (3,650 of 102,878) of patients in the current report versus 5.5% (744 of 13,473) from GENIE. *MDM2* amplification was most commonly seen in patients with liposarcoma (63.6% [332 of 522] in the current report and 69.1% [47 of 68] from GENIE); gallbladder, adenocarcinoma (11.1% [62 of 554] in the current report and 17.5% [seven of 40] from GENIE); sarcoma, not otherwise specified (10.7% [103 of 955] in the current report and 25.0% [four of 16] from GENIE); urothelial carcinoma (10.4% [198 of 1,898] in the current report and 10.7% [48 of 446] from GENIE); and lung, adenosquamous carcinoma (9.8% [17 of 173] in the current report and 9.0% [one of 11] from GENIE).

Among the 102,878 diverse cancers studied, *MDM2* amplification was identified in 3,650 (3.5%). *MDM2* amplification was most commonly seen among liposarcoma (63.6% [332 of 522]) followed by gallbladder, adenocarcinoma (11.1% [62 of 554]); sarcoma, not otherwise specified (10.7% [103 of 955]); and urothelial carcinoma (10.4% [198 of 1,898]; Table 1). *MDM2* amplification was not found among anaplastic and papillary carcinoma of thyroid (zero of 166 and 376, respectively) and uncommonly among adenocarcinoma of the appendix, rectum, and colon (0.23% [one of 440], 0.28% [four of 1,448], and 0.33% [28 of 8,562], respectively; Data Supplement). In comparison, according to the GENIE database (total, 13,473 samples), *MDM2* amplification has been reported in 5.5% (744 of 13,473) of diverse cancers, including liposarcoma (69.1% [47 of 68]); gallbladder, adenocarcinoma (17.5% [seven of 40]); sarcoma, not otherwise specified (25.0% [four of 16]); and urothelial carcinoma (10.7% [48 of 446]; Fig 1).

### Genomic Co-Alterations Associated With *MDM2* Amplification

Among 3,650 patients with *MDM2* amplification, 99.0% (3,613) were found to have genomic co-alterations (Data Supplement). Frequently co-altered genes were *CDK4* (43.6% [1,591 of 3,650]), *FRS2* (40.8% [1,491 of 3,650]), *TP53* (20.1% [733 of 3,650]), and *CDKN2A* (18.2% [665 of 3,650]; Data Supplement). In contrast, among patients with wild-type *MDM2*, *CDK4* and *FRS2* alterations were rare compared with those with *MDM2* amplification (1.2% and 0.20%, respectively; both *P* < .001). *TP53* alterations were more common in patients with *MDM2* wild type (53.6%; *P* < .001; Data Supplement). When co-alterations are grouped into specific pathways, cell cycle–associated genes were most commonly co-altered (68.5% [2,502 of 3,650]) followed by tyrosine kinase–associated genes (37.9% [1,385 of 3,650]), *PI3K* signaling–associated genes (25.4% [926 of 3,650]), *TP53-*associated genes (24.9% [910 of 3,650]), and *MAPK* signaling–associated genes (23.6% [863 of 3,650]). Although uncommon, mismatch repair genes and *PD-L1* amplification were co-altered in 2.2% (79 of 3,650) of patients (Table 2).

**Table 2.**
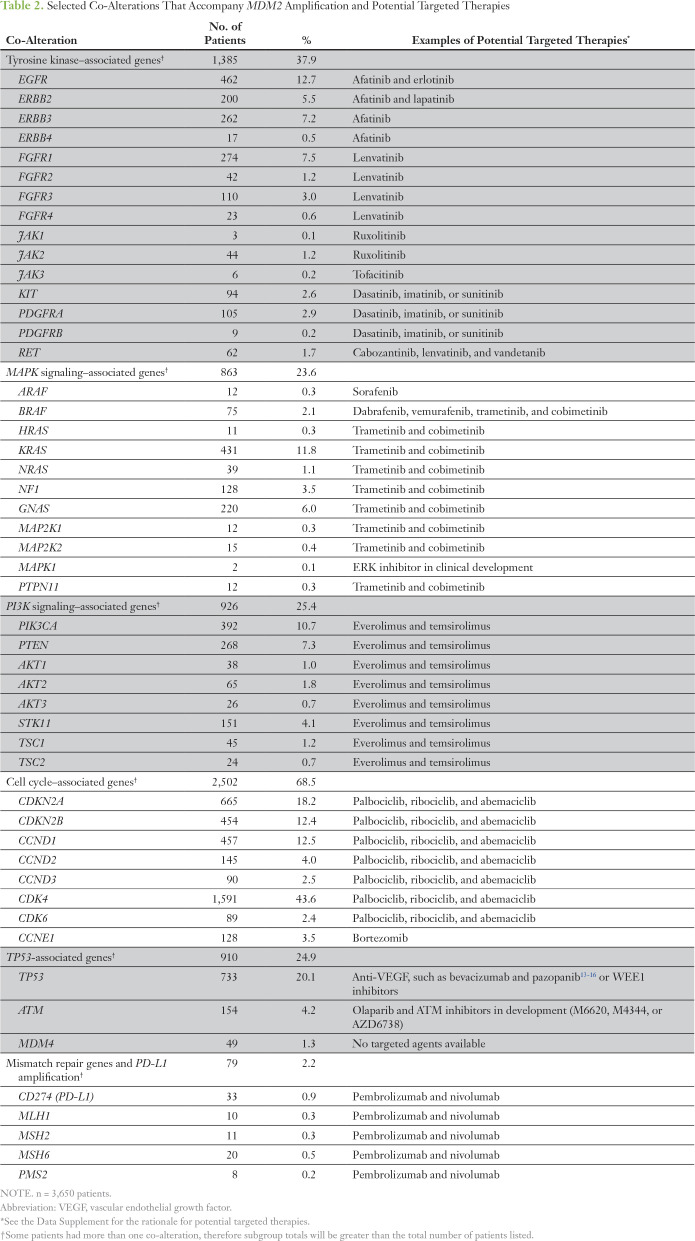
Selected Co-Alterations That Accompany *MDM2* Amplification and Potential Targeted Therapies

### Potential Cognate-Targeted Therapies for Genomic Co-Alterations Associated With *MDM2* Amplification

Among 3,650 patients with *MDM2* amplification, the median number of alterations per patient was six (range, one to 25); 96.9% (3,536) had one or more co-alterations targetable with a Food and Drug Administration–approved agent (either on or off label). An additional 0.7% (27 of 3,650) of patients had one or more co-alterations targetable with investigational agents. Altogether, 97.6% (3,563 of 3,650) of patients had genomic co-alterations actionable with either a Food and Drug Administration–approved or investigational agent; the median number of potentially targetable genomic co-alterations was three (range, zero to 17; Data Supplement).

### Association of *MDM2* Amplification and Mutational Burden

Among diverse cancers (N = 102,878), high TMB status was significantly less frequent in patients with *MDM2* amplification than in the *MDM2* wild-type population (2.9% [105 of 3,650] *v* 6.5% [6,492 of 99,228], respectively; *P* < .001). A similar difference also was observed in the GENIE data set (frequency of high TMB among *MDM2* amplification *v MDM2* wild type, 3.4% [25 of 744] *v* 5.6% [714 of 12,729], respectively; *P* = .008).

**Fig 2.**
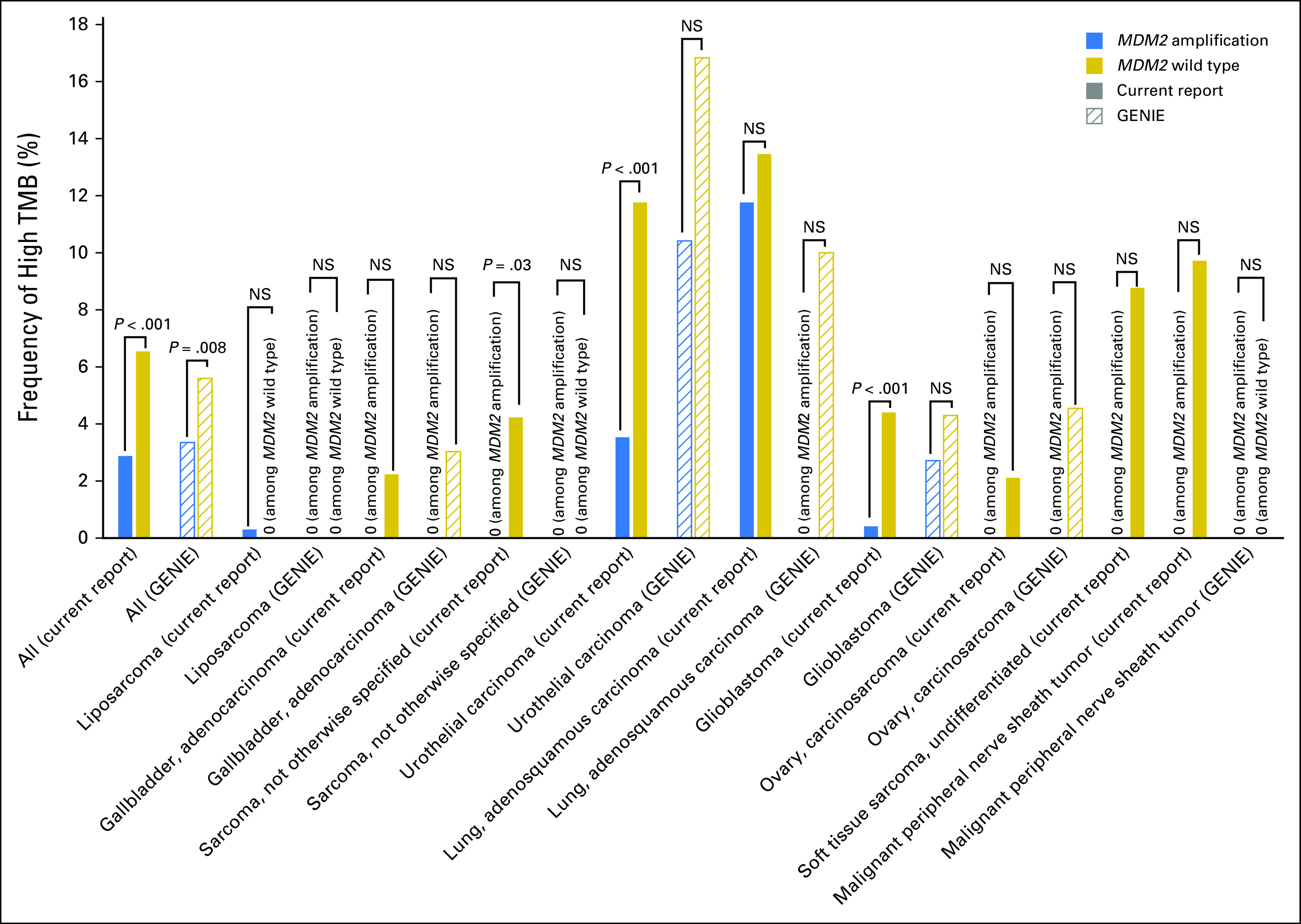
Association between *MDM2* amplification and tumor mutation burden (TMB). Among patients with *MDM2* amplification (n = 3,650), 2.9% (105) had high TMB, 23.3% (852) had intermediate TMB, and 73.8% (2,693) had low TMB. Among diverse cancers (N = 102,878), high TMB status was significantly less frequent in patients with *MDM2* amplification than in those with *MDM2* wild type (2.9% [105 of 3,650] *v* 6.5% [6,492 of 99,228]; *P* < .001). The Genomics Evidence Neoplasia Information Exchange (GENIE) data set also showed a similar observation (frequency of high TMB among *MDM2* amplification *v MDM2* wild type, 3.4% [25 of 744] *v* 5.6% [714 of 12,729], respectively; *P* = .008). In the current data set, certain cancers with *MDM2* amplification were significantly less associated with high TMB than with *MDM2* wild type (sarcoma, not otherwise specified, 0% [zero of 103] *v* 4.2% [36 of 852], respectively [*P* = .03]; urothelial carcinoma, 3.5% [seven of 198] *v* 11.8% [200 of 1,700], respectively [*P* < .001]; glioblastoma, 0.4% [one of 244] *v* 4.4% [120 of 2,725], respectively [*P* < .001]); these subsets did not show significant differences in GENIE, but the number of patient samples in GENIE was considerably smaller (Data Supplement). NS, not significant.

In the current data set, subsets of cancer with *MDM2* amplification also were associated with less-frequent high TMB status compared with *MDM2* wild type (sarcoma, not otherwise specified, 0% [zero of 103] *v* 4.2% [36 of 852], respectively [*P* = .03]; urothelial carcinoma, 3.5% [seven of 198] *v* 11.8% [200 of 1,700], respectively [*P* < .001]; glioblastoma, 0.4% [one of 244] *v* 4.4% [120 of 2,725], respectively [*P* < .001]; Fig 2; Data Supplement). These differences in patient subsets were not seen in the GENIE data set perhaps because GENIE had considerably fewer patients (approximately 13% of the patients in our data set).

Among patients with *MDM2* amplification (n = 3,650), *TP53* was co-altered in 733. Most patients with *MDM2* amplification and wild-type *TP53* had low TMB (98.4% [2,817 of 2,917]); among patients who harbored both *MDM2* amplification and *TP53* alteration, 55.3% (405 of 733) had low TMB (*P* < .001).

### *MDM2* Amplification as a Potential Marker for Hyperprogression With Immune Checkpoint Inhibitors

**Fig 3.**
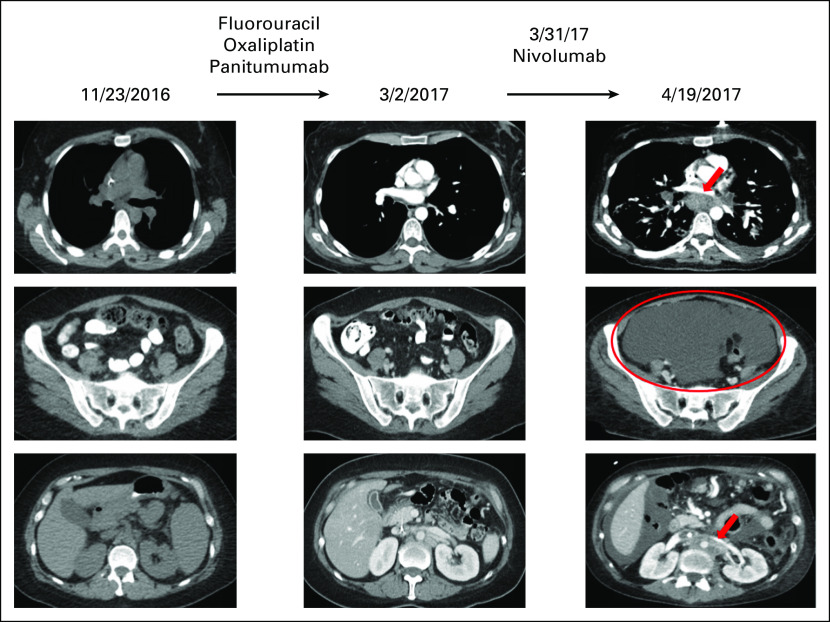
Hyperprogression in a patient with *MDM2* amplification treated with an anti–PD-1 checkpoint inhibitor.^22^ A 36-year-old woman presented with worsening dysphagia and anemia. Additional work-up revealed adenocarcinoma of the gastro-esophageal junction, stage IIIC. The patient was initially started on combination chemotherapy with epirubicin, oxaliplatin, and capecitabine with persistent lymphadenopathy. Therapy was switched to fluorouracil, oxaliplatin, and panitumumab with overall stable disease; however, the patient had persistent subcentimeter lymphadenopathy (left and middle). The regimen was then switched to nivolumab (anti–PD-1 inhibitor). Within 3 weeks, the patient showed marked clinical deterioration, and imaging showed rapid progression in mediastinal and retroperitoneal lymph nodes as well as emerging massive ascites (right). Pace of progression increased by 6.4-fold, tumor burden increased by 460% compared with pre-immunotherapy imaging, and time to treatment failure was 3 weeks (hyperprogression after immunotherapy previously defined as more than a two-fold increase in progression pace, a > 50% increase in tumor burden compared with pre-immunotherapy imaging, and a time to treatment failure < 2 months^22^). Therapy was then changed to fluorouracil, oxaliplatin, and trastuzumab, but the patient died 1.5 months after nivolumab was administered. Molecular profiling of the primary tumor revealed multiple alterations, including *MDM2* amplification. Other alterations were *ERBB3*, *ARAF*, *CDK4*, and *EGFR* amplifications and alterations in *PIK3CA*, *FRS2*, *GLI1*, and *IKZF1*. Tumor mutation burden was low and microsatellite stable. PD-1 and PD-L1 status by immunohistochemistry were not evaluated.

We have previously reported that *MDM2* amplification can be associated with hyperprogression after treatment with anti–PD-1/PD-L1 agents.^22^ We describe herein a 36-year-old woman (not previously reported) with adenocarcinoma of the gastro-esophageal junction who had stable disease (SD) while receiving second-line therapy with fluorouracil, oxaliplatin, and panitumumab (Fig 3, left and middle). For persistent, subcentimeter lymphadenopathy, the regimen was switched to nivolumab (anti–PD-1 inhibitor). The patient had rapid progression in the mediastinal and retroperitoneal lymph nodes as well as emergent massive ascites (time to treatment failure, 3 weeks; pace of progression increased by 6.4-fold compared with the 4 months before the start of checkpoint blockade, and tumor burden increased 460% compared with pre-immunotherapy imaging; Fig 3). The patient succumbed to disease 1.5 months after nivolumab administration. Molecular profiling of the primary tumor revealed alterations, including amplifications in *MDM2*, *ERBB3*, *ARAF*, *CDK4*, and *EGFR* and alterations in *PIK3CA*, *FRS2*, *GLI1*, and *IKZF1*. TMB was low and microsatellite stable. PD-1 and PD-L1 immunohistochemistry was not evaluated.

## DISCUSSION

We and others recently demonstrated that approximately 9% of patients treated with PD-1/PD-L1 checkpoint blockade exhibit a paradoxical acceleration in tumor progression (designated as hyperprogression). This phenomenon associates with *MDM2* amplification.^22-24^ Therefore, caution is needed in treating patients who harbor *MDM2* amplification with checkpoint inhibitors, and a thorough understanding of the *MDM2* alteration landscape is clinically important. Therefore, we describe the genomic backdrop of *MDM2* amplification among 102,878 patients with diverse malignancies. Overall, *MDM2* amplification was found in 3.5% (3,650 of 102,878) of cancers (a number similar to that in the GENIE database [5.5% (744 of 13,473)]; Fig 1). *MDM2* amplification most commonly has been seen in patients with liposarcoma (63.6% [332 of 522])^5^ but discerned in a subset of most tumor types, albeit at different frequencies (Table 1; Data Supplement). Certain diagnoses (eg, anaplastic and papillary thyroid cancer) were not associated with *MDM2* amplification, and this anomaly was rare in acute myelocytic leukemia (one of 1,006 patients; Data Supplement).

An understanding of the comprehensive landscape of *MDM2* amplification also is therapeutically relevant because MDM2 inhibitors are in clinical development (Data Supplement). Clinical activity of MDM2 inhibitors among unselected diverse cancers has been limited^17-19^; however, occasional responses have been observed in individuals selected for wild-type *TP53*.^20,21^ The low response rate with single-agent MDM2 inhibitors may be due to the lack of patient selection for *MDM2* amplification or to co-altered genes (Data Supplement). Accumulating evidence has suggested that the matched targeted therapy approach can demonstrate better clinical outcomes than a nonmatched approach, but this implies the need to select patients for the relevant aberration.^34-37^ Wagner et al^21^ showed that responses with MK-8242 (MDM2 inhibitor) were exclusively observed in patients with liposarcoma (RR, 11.1% [three or 27]) whose molecular hallmark includes *MDM2* amplification^5^ (nonliposarcoma; RR, 0% [zero of 14]). On the other hand, even in a disease such as liposarcoma where > 60% of patients have *MDM2* amplification, the RR is relatively low, which may be due to, as mentioned previously, the presence of co-alterations. Indeed, the 12q13-15 amplicon on which *MDM2* resides is large (but discontinuous); *CDK4* and *FRS2* reside on the amplicon and frequently are co-amplified with *MDM2*^38^ but are rarely abnormal in patients without *MDM2* amplification (Data Supplement).

**Fig 4.**
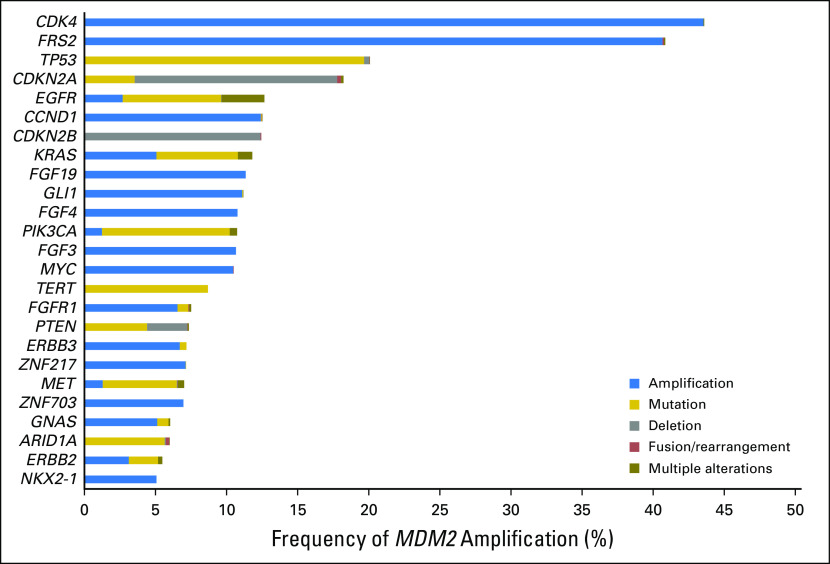
Genomic co-alterations associated with *MDM2* amplification (n = 3,650). The most common co-alterations associated with *MDM2* amplification were *CDK4* (43.6% [1,591 of 3,650]), *FRS2* (40.8% [1,491 of 3,650]), *TP53* (20.1% [733 of 3,650]), *CDKN2A* (18.2% [665 of 3,650]), and *EGFR* (12.7% [462 of 3,650]; Data Supplement).

In keeping with the notion that co-alterations are important, we also assessed the alterations that co-occurred with *MDM2* amplification. The majority of *MDM2-*amplified tumors harbored co-alterations (99% [3,613 of 3,650]); the median number of alterations per patient was six (range, one to 25; Data Supplement). The most common co-alterations were indeed *CDK4* and *FRS2* amplification (Fig 4); therefore, the targeting of *MDM2* amplification alone may be insufficient to achieve satisfactory antitumor activity (Data Supplement). Additional clinical trials that investigate the feasibility and efficacy of matched targeted combination strategies are required. Because *FRS2* and *CDK4* are on the *MDM2* amplicon, the targeting of them may warrant specific study.

Of note, we also observed that *TP53* alterations were not mutually exclusive with *MDM2* amplification, as previously reported.^5,6^ Although *TP53* alterations were less commonly seen in patients with *MDM2* amplification compared with wild type (Data Supplement), *TP53* alterations were observed in 20.1% (733 of 3,650; Fig 2; Data Supplement). Because *MDM2* amplification suppresses the function of p53, co-alteration of *TP53* with *MDM2* amplification suggests a noncanonical, p53-independent role for MDM2 in tumorigenesis. One of the proposed noncanonical roles of MDM2 is to facilitate angiogenesis.^39^ Zhou et al^10^ reported that MDM2-overexpressed/*TP53*-null cancer cells are associated with increased VEGF mRNA expression compared with MDM2-negative/*TP53*-null cells. Furthermore, Lakoma et al^11^ showed that pharmacologic inhibition of MDM2 is associated with a decrease in hypoxia-inducible factor 1α and VEGF expression in cancer cell lines. *TP53* alterations also have been reported to be associated with increased VEGF expression in preclinical models as well as in patients with lung adenocarcinoma.^12,13^ Clinically, patients with cancer with *TP53* alterations have been shown to experience longer progression-free survival (PFS) and a higher rate of SD of ≥ 6 months/partial and complete remission with bevacizumab (anti-VEGF antibody)–containing regimens compared with non-bevacizumab–containing regimens (median PFS, 11.0 *v* 4.0 months [*P* < .001]; SD ≥ 6 months/partial and complete remission, 31% *v* 7% [*P* ≤ 0.01]).^15,16^ *TP53* alteration status also predicted longer PFS among patients with sarcoma treated with pazopanib (multikinase inhibitor that targets the VEGF receptor; hazard ratio, 0.38; *P* = .036).^14^ Thus, the harboring of either *MDM2* amplification or *TP53* alteration may lead to enhanced angiogenesis, which may be susceptible to anti-VEGF therapies. Additional investigation is warranted.

Mismatch repair genes and *PD-L1* amplification were co-altered in 2.2% (79 of 3,650) of the patients with *MDM2* amplification (Table 2). Tumors with mismatch repair deficiency or *PD-L1* amplification have been associated with remarkable responses to immune checkpoint inhibitors.^40-42^ On the other hand, we have previously reported that *MDM2* amplification and *EGFR* alterations (both of which were discerned in the current patient example) were significantly associated with hyperprogression when anti–PD-1/PD-L1 agents were used.^22^ In this prior report, all four patients with hyperprogression and available data had negative PD-L1 expression; the one patient with available data had high TMB. In the current study, we depict an individual with gastric cancer that harbored *EGFR* as well as *MDM2* amplification who had indolent disease; the patient, however, showed explosive progression after being given the anti–PD-1 inhibitor nivolumab (Fig 4). Whether patients who have both *PD-L1* amplification (a marker of sensitivity to checkpoint inhibitors in Hodgkin disease^43^) and *MDM2* amplification would respond to checkpoint blockade is unclear. Of note, tumors that harbor *MDM2* amplification had significantly lower rates of high TMB than *MDM2* wild-type tumors (2.9% [105 of 3,650] *v* 6.5% [6,492 of 99,228]; *P* < .001). Because high TMB correlates with checkpoint blockade responsiveness,^44,45^ this observation may partially explain resistance to PD-1/PD-L1 inhibitors in *MDM2*-amplified tumors but does not clarify the mechanism that underlies the correlation between *MDM2* amplification and hyperprogression. Furthermore, how patients whose tumors have high TMB as well as *MDM2* amplification would fare on checkpoint inhibitors is unclear; however, one of our previously reported patients with *MDM2* amplification who demonstrated hyperprogression with anti–PD-L1 immunotherapy had a high TMB.^22^ Finally, an issue that merits prospective exploration is how a combination of MDM2 and checkpoint inhibitors would affect the risk of hyperprogression in patients whose cancers bear an *MDM2* amplification.

The current study has several limitations. First, the data set was de-identified, which limited the analyzable correlates; thus, clinical questions such as the frequencies of *MDM2* amplification that depend on the disease state (early stage *v* metastatic and recurrent disease) could not be evaluated. Second, because the number of patients in each cancer type was based on the number of samples sent for NGS by the treating physicians, sample size bias is possible. Finally, the cancer diagnosis was annotated on the basis of the submitting physician’s description. Despite these limitations, the current study provides, to our knowledge, the largest and most comprehensive analysis of *MDM2* amplification in diverse malignancies to date.

In summary, we have interrogated 102,878 patients with diverse cancers and demonstrated that amplification of *MDM2* is found in 3.5% (3,650) of tumors. The majority of cancer types included a subgroup of patients, albeit small, with *MDM2* amplification. Most patients (99.0% [3,613 of 3,650]) harbored co-alterations with *MDM2* amplification (97.6% potentially targetable). Although infrequent, mismatch repair genes and *PD-L1* amplification also were co-altered in 2.2% (79 of 3,650) of patients. In addition, high TMB was significantly less common among patients with *MDM2* amplification. This study suggests that *MDM2* amplification is found in a subset of most cancer diagnoses and that optimization of targeted therapy against MDM2 and immunotherapy might require relevant combinations of drugs.
